# Islet-Resident Dendritic Cells and Macrophages in Type 1 Diabetes: In Search of Bigfoot’s Print

**DOI:** 10.3389/fendo.2021.666795

**Published:** 2021-04-12

**Authors:** Henner Zirpel, Bart O. Roep

**Affiliations:** Department of Diabetes Immunology, Diabetes & Metabolism Research Institute at the Beckman Research Institute, City of Hope National Medical Center, Duarte, CA, United States

**Keywords:** macrophage, dendritic cell, islets of Langerhans, innate immunity, beta-cell stress, autoimmune diseases

## Abstract

The classical view of type 1 diabetes assumes that the autoimmune mediated targeting of insulin producing ß-cells is caused by an error of the immune system. Malfunction and stress of beta cells added the target tissue at the center of action. The innate immune system, and in particular islet-resident cells of the myeloid lineage, could function as a link between stressed ß-cells and activation and recognition by the adaptive immune system. We survey the role of islet-resident macrophages and dendritic cells in healthy islet homeostasis and pathophysiology of T1D. Knowledge of islet-resident antigen presenting cells in rodents is substantial, but quite scarce in humans, in particular regarding dendritic cells. Differences in blood between healthy and diseased individuals were reported, but it remains elusive to what extend these contribute to T1D onset. Increasing our understanding of the interaction between ß-cells and innate immune cells may provide new insights into disease initiation and development that could ultimately point to future treatment options. Here we review current knowledge of islet-resident macrophages and dendritic cells, place these in context of current clinical trials, and guide future research.

## Introduction

Type 1 diabetes is characterized by the loss of insulin-producing ß-cells in pancreatic islets of Langerhans leading to insulin shortage. This loss is caused by an autoimmune mediated attack, in which ß cell specific CD8^+^ T-cells are the ultimate effectors. In past decades ß-cells were deemed “innocent victims” of this autoimmune attack. Consequently, intervention therapies focused to suppress the adaptive immune system, but showed limited success (1). Plausibly, the cause of T1D is not only due to an erroneous immune system and involves additional pathophysiological reasons. Research shifted toward ß-cells provoking autoimmunity, changing our view of T1D immunopathogenesis in which stressed ß-cells trigger an autoimmune attack in a predisposing genetic and immunological environment (2, 3). An important gap in knowledge is what kicks off this process and what connects the adaptive immune system and ß-cells. Pancreatic islets are complex micro-organs. Besides hormone releasing cells, resident antigen presenting cells (APCs) of the myeloid lineage and innervating neurons are present. In spite of their footprint in islets, little is still known about resident myeloid cells and whether these cells play any role in health or disease, alike the snowprint of Bigfoot, the mystical legend that most scientist consider to be a misidentification. We propose that myeloid APCs are the missing link between distressed ß-cells and the adaptive immune system. We focused attention to islet-resident myeloid cells and investigated their possible role as connectors bridging ß-cells and adaptive immunity.

## T1D as a Disease of the Adaptive Immune System

T1D is a disease of the adaptive immune system (4). The best tool to predict T1D onset is screening for islet autoantibodies. These can be directed against a range of different islet antigens, including insulin, glutamate decarboxylase, zinc transporter 8 and insulinoma antigen-2 (5). Their appearance follows activation of T-cells and depends on poorly understood interactions between the environment, genetic factors and the immune system in a process that can range from months to years before clinical manifestation of T1D. However, positive autoantibody testing does not necessarily imply onset of disease and proof of a direct role of islet autoantibodies in beta-cell destruction is still lacking (6). Islet autoreactive CD4^+^ and CD8^+^ T-cells are present in islets, blood and lymph nodes (7). Distressed islets of T1D patients display increased HLA class I on the surface of endocrine cells, apparently preceding insulitis and facilitating autoreactive CD8^+^ mediated ß-cell targeting. Both islet autoantibodies and islet-autoreactive T-cells indicate a break in immune tolerance and identify the adaptive immune system as essential component in the autoimmune process leading to loss of beta-cells.

Auto-reactive T-cells evade thymic education in both healthy individuals and patients with T1D (8). Regulatory T-cells are critical in maintaining tolerance and are present in similar numbers in healthy and diseased individuals but display reduced regulatory potential in patients (9). An imbalance between immune regulation and activation in favor of islet autoimmunity is evident in T1D (8). Yet, why T-cell becomes activated and what role beta-cells and the innate immune system may play in this imbalance remains largely unknown.

## T1D as a Disease of the Beta-Cell

The idea of T1D being a disease of the adaptive immune system has become challenged in the past decade (4). Several observations point toward additional key players. Research shifted toward the ‘victim’ target tissue and increasing evidence places ß-cells at the center of initiation.

**Figure 1:**
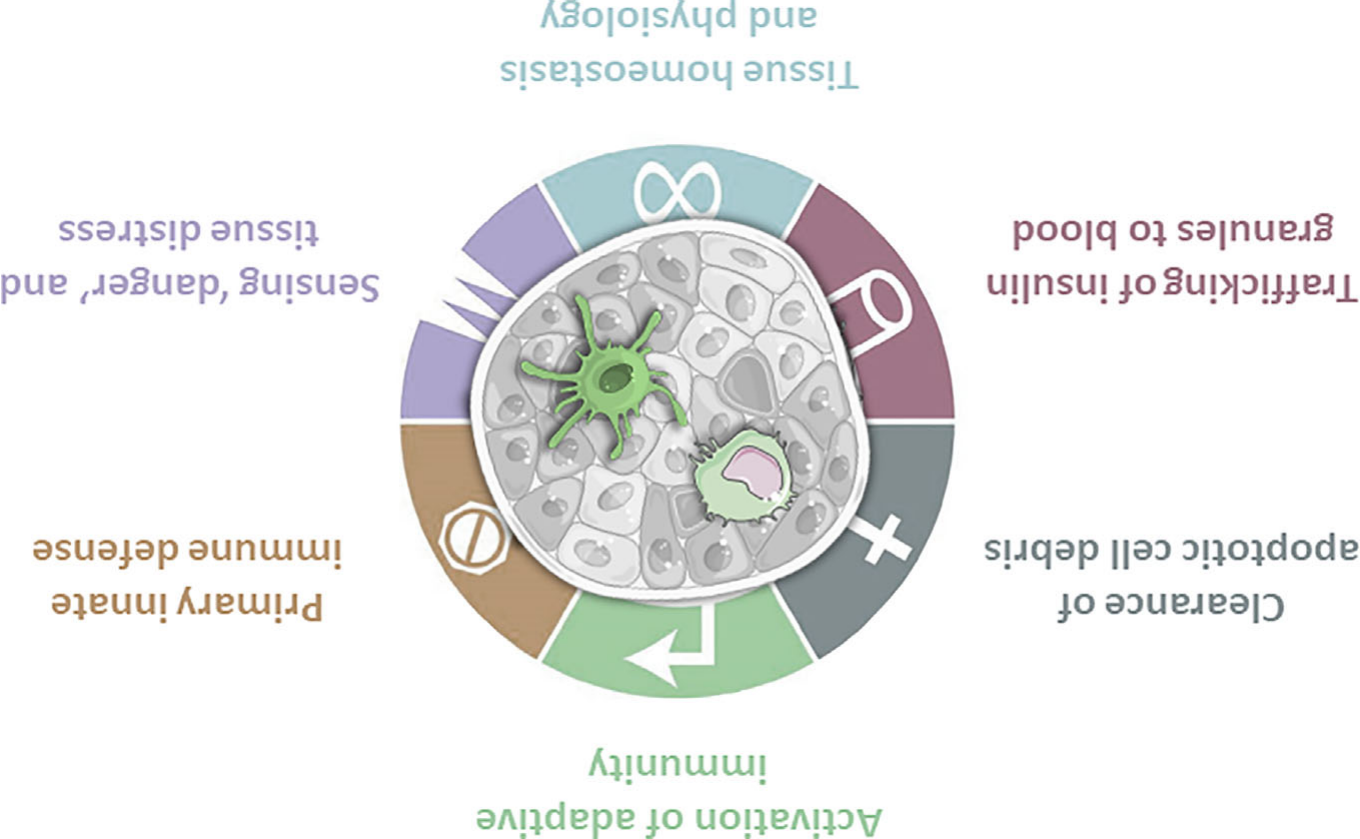
Antigen presenting cells are present in islets of Langerhans. These innate immune cells fulfill a wide range of tasks. Macrophages play a crucial role in tissue homeostasis and physiology by expressing tissue remodeling cytokines. Due to constant sampling of the surrounding environment they clear apoptotic cell debris, but also sense danger signals and tissue distress. Obtained granules are then trafficked to the blood. In case of invading pathogens macrophages and dendritic cells are first line of defense. Dendritic cells are mainly involved in screening for danger signals and subsequent activation or regulation of the adaptive immune system.

ß-cells are highly specialized to produce large quantities of insulin (10). This specialization is at expense of reduced defense mechanisms and pronounced stress sensibility. Cellular stress could result from constantly increased demand of insulin. Pancreas sizes seem to matter in T1D (11). A smaller pancreas implies reduced numbers of ß-cells, which subsequentially increases the metabolic burden on islets (12). Beside reduced pancreas size, other factors such as viral infections or inflammatory milieu have been suggested as stressors (13, 14). Pancreata from T1D donors showed ß-cells under increased intra-cellular stress during insulitis as indicated by markers of endoplasmic reticulum stress, such as CHOP, BIP and XBP-1 (15). ß-cell stress may trigger adaptive immunity but this requires involvement of the innate immune system, since activation of islet auto-reactive T-cells only occurs following priming by dendritic cells due to presentation of immunogenic islet peptides (**Figure 1**). Research on stressed ß-cells revealed various mechanisms for the generation of new auto-immune peptides (neoantigens) not present during thymic education, selection and formation of the immune system. Post-translational modifications add to variety of the proteome and modified peptides might be recognized as neo-epitopes (16–18). Other neoantigens include so-called hybrid peptides joining peptides fragments of two islet proteins, which stimulate T-cells found in islets of T1D patients (19, 20). Neoantigens can also occur by erroneous translation, leading to defective ribosomal proteins (DRiP), or by alternative splicing (21–23). DRiPs can be generated by ribosomal complex skipping of the canonical start codon and instead initiation at a start codon within an alternative reading frame. This whole set of ß-cell released stressors points toward T1D being a disease of the adaptive immune system as well as ß-cells, where distressed ß-cells change their faces and prime the immune system.

## The Innate Immune System as a Connector

**Figure 2:**
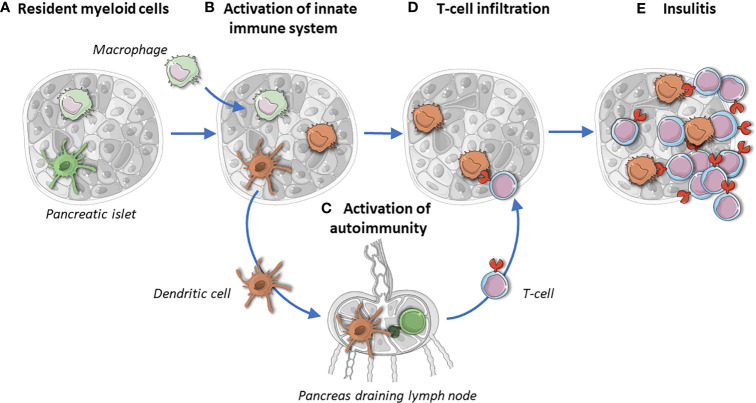
Islets resident myeloid cells maintain tissue homeostasis and protect islets **(A)**. In T1D, macrophages infiltrate islets and their ratio changes toward a pro-inflammatory phenotype **(B)**. Upon activation, islet-resident dendritic cells migrate to pancreatic draining lymph nodes and activate naïve T-cells **(C)**. Activated T cells infiltrate islets and CD4^+^ T cells scan for islet autoantigens taken up, processed and presented by macrophages and dendritic cells **(D)**. Insulitic auto-reactive CD8^+^ T cells target ß-cells **(E)**. The missing link in **(D, E)** is the place of the elusive dendritic cells: are they still there, if so, how many and what do they look like? Brown stands for activated myeloid cells.

Macrophages and dendritic cells are professional APCs and the most extensively studied myeloid cells. They are present in islets and accumulate there during disease progression (**Figures 1**, **2**) (24). One of two major classes of APCs are dendritic cells (DCs). Immature DCs are tolerogenic (25). Conventional dendritic cells (cDCs) are strong APCs that activate naïve T-cells once they mature upon stimulation, while plasmacytoid dendritic cells (pDCs) secrete large amounts of proinflammatory interferons. DCs play a crucial role in maintaining immune tolerance and preventing tissue-specific autoimmunity, which harbors great therapeutic potential.

Macrophages can be divided into pro-inflammatory ‘M1’ and anti-inflammatory ‘M2’ macrophages based on their phenotype (26). However, this strict classification is changing into tissue and microenvironment specific flavors. Based on their local microenvironment, monocytes can differentiate into different subtypes of macrophages and fully differentiated macrophages are able to change their phenotype when transferred into other tissues (27). Additionally, changes are observed in enhancer landscape and gene expression profiles in different tissue-resident macrophages (28).

Having auto-reactive T-cells on one side and stressed ß-cells on the other raises the question how these two players interact. APCs characteristically infiltrate and monitor different tissues. They become activated upon recognition of pathogen- or danger-associated molecular patterns, resulting in different responses, such as migration (29). DCs migrate after uptake of antigen from tissue to draining lymph nodes for antigen presentation and subsequent activation of antigen specific T-cells (**Figures 1** and **2**) (30, 31). Therefore, DCs could function as physical activators of T-cells in T1D (**Figure 2**). Besides DCs, macrophages play a crucial role in tissue homeostasis and antigen presentation toward approaching T-cells (**Figure 1**). The observation that transplantation of islets is more successful upon APC depletion strengthens the idea that resident APCs play contribute to onset (32, 33). If APCs are the missing link between ß-cells and the innate immune system, it is necessary to determine their individual role in a spatial manner.

## Myeloid Cells in Circulation in Health and Disease

Given that APCs play a crucial role in T1D onset by connecting ß-cells to the adaptive immune system, it is worth to assess differences in APCs between healthy individuals and T1D patients. Studies in NOD mice indicate altered numbers of DCs in blood and thymus compared to control mice (34, 35) and a DC subtype analysis reported an imbalance toward CD8α^-^ DCs (36, 37). Several studies claim possible variation in humans. Yet, whether DC numbers are increased, decreased, or remained stable and whether this happens before, during, or after onset is inconsistent (38–43). These inconsistences might relate to the notion that most studies focused mainly on monocyte derived DCs rather than *bona fide* DCs. Besides quantitative changes, functional differences, such as reduced phagocytic capabilities or increased HLA-DR expression in T1D have been reported (44).

Monocyte derived tolerogenic DCs (tolDcs) from patients under sub-optimal glycemic control display reduced tolerogenic capabilities compared to those from patients under optimal control (45–48). However, this glycemia dependent difference may not necessarily be a general difference between health and disease, and could be a consequence, rather than causally related to T1D immunopathogenesis. We recently showed that tolDCs generated from T1D patients’ blood induce immune tolerance indifferently from those from healthy individuals, proving that they still possess their immune-regulatory capacity (47).

## Resident Myeloid Cells in Health and Disease

Besides circulating APCs, the role of resident APCs must be evaluated, especially since these are the first sensors of any changes in islets (**Figure 1**) (49). Islets contain macrophages as shown by staining for CD68 using imaging mass cytometry (50–52). They were present in low numbers and numbers were greater before and after onset of disease (52). However, it remains unclear whether infiltrating macrophages differ from resident ones, whether resident macrophages change, and whether they affect, or are affected by, the islet microenvironment in T1D.

Studies from NOD mice show that the vast majority (up to 98%) of APCs are macrophages, while inconsistencies exist about the presence of other APCs, such as DCs (53–55). Analysis of resident macrophages in NOD mice classifies them as cells with a mixed M1/M2 phenotype, polarizing toward M1, as indicated by transcripts of IL1b and TNFα. During pancreas development immature macrophages enter the islets and mature by week 4 of age, as measured by MHC II (53). Afterward, they are self-maintaining with low infiltration of immature macrophages or monocytes (53). The occurrence of mature APCs by week 4 is particularly interesting, since NOD mice develop insulitis soon after. NOD mice did not develop diabetes in absence of resident macrophages (56). Depletion of islet-resident APCs at 8 weeks of age resulted in a complete disappearance of lymphocytes from the pancreas. Upon reappearance of DCs and macrophages, lymphocytes reappeared (57). Ex vivo depletion resulted in a reduced release of pro-inflammatory cytokines such as IL-6, IP-10, and G-CSF (58). Interestingly, T-cells from macrophage-depleted NOD mice were unable to induce diabetes upon transfer into NOD.*scid* mice (59–61).

Beside their function as APCs, macrophages play a critical role in tissue development and remodeling (**Figure 1**), where they promote proliferation of ß-cells by creating a favorable microenvironment and upregulation of SMAD7 (62–65). During pancreas development in mice macrophages were present at increased numbers that declined until weaning (66). Curiously, lymphocyte infiltrates consisting of T-cells with some macrophages and DCs were observed in human fetal and neonatal pancreata (67). Lack of macrophages as in osteopetrotic *op/op* mice (CSF1^-/-^), or due to chemical or antibody depletion, resulted in reduced pancreas size and vasculature, supporting a crucial role of resident macrophages, given that T1D patients also display reduced pancreas sizes (11, 68). With regards to vascularization, human islets from T1D patients display lower levels of vascular endothelial growth factor-A (VEGF-A) (58). VEGF-A is produced by ß-cells and seems to play a role in the development of islet vasculature, in ß-cell function, and in macrophage mediated ß-cell proliferation (69–71). Since resident macrophages are located in close contact to vasculature it seems plausible that some crosstalk between ß-cells, macrophages, and vasculature exist (72). However, this interplay remains elusive and warrants further studies. Macrophages might also directly induce beta-cell destruction by the synthesis of proinflammatory cytokines and reactive oxygen species, which lead to the so-called ‘Copenhagen model’ that put macrophages at the heart of islet inflammation and beta-cell destruction (73). While support of cytokine-mediated beta-cell toxicity was obtained in rodents, this did not hold for human beta-cells that proved far more resistant to cytokines (requiring a 100-fold larger dose than is not even feasible pathologically) and much better at dealing with oxygen radicals than rodents (74).

In marked contrast to mice, macrophages in humans only make for half or less of resident APCs and their phenotype was reported to be mixed M1/M2 (TNF, IL1b, IL6, IL10, with release of additional tissue remodeling cytokines MMP2, MMP9) (51, 75). Alike macrophages from other tissues that maintain tissue homeostasis by sensing hyperosmolarity, metabolic stress, hypoxia and ECM components, islet-resident macrophages sense their surrounding by detecting extracellular ATP concentrations *via* purigenic receptors, resulting in an increased concentration of intracellular Ca^2+^ levels (72, 75). Since ATP concentrations correlate with insulin levels, macrophages can sense ß-cell function.

In addition to microenvironment sensing, resident APCs constantly probe their surroundings. Islet-resident macrophages engulf vesicles released from ß-cells, a process taking place over a short distance, process and present these (76–78). Importantly, these granules contain immunogenic peptides, which can be recognized by auto-reactive T-cells that had escaped thymic education (79–82). Such peptides can be taken up by DCs and their presence in draining lymph nodes is confirmed, which can result in activation of the innate immune system (**Figure 2**). Subsequent, targeting of immunogenic peptide presenting APCs by autoreactive CD4^+^ T-cells supports this process of initiation and strengthens macrophages’ potential role in onset (83, 84).

But where is ‘Bigfoot’, the dendritic cell in human islets? While mouse studies suggest that the myeloid compartment in islets is up to 98% consisting of macrophages, the rare studies on human islets pointed that 50% of leukocytes at best were macrophages, while the other 50% was ignored. We contend that islet DCs are important candidates to be identified and characterized, given their key role in regulating immune activation and modulation (**Figure 2**). Curiously, studies in both mice and men thus far have been biased to either macrophages or DCs. This leaves a significant opportunity to study the role of islet DCs in health and disease.

## Chicken or Egg

The above presented data point toward differences in myeloid cells between mice and men, between health and T1D, between different individuals and between neighboring islets. Even though genetic differences in the myeloid lineage exist, phenotypic alterations might not necessarily be present from the beginning (48). Instead, they might appear only in an altered microenvironment, such as in inflamed islets or hyperglycemia. The microenvironment plays a crucial role for macrophages, since these cells possess high microenvironment-dependent plasticity, which results in change of their phenotype (27). A stressed microenvironment caused by distressed ß-cells due to infection or other perturbations (metabolic, inflammatory) could lead to genetically prone malfunctioning of macrophages, or indeed be caused by these innate immune cells. Subtle changes in the microenvironment could occur over years that have skipped attention. The role of the microenvironment on macrophage phenotype is supported by recent findings showing that microenvironment alters infiltrating macrophages after diabetes onset (85). Such changes might also occur in healthy individuals but be better compensated.

Another question arising is whether APCs engulf, process, and present antigen in a different way in diabetes prone subjects. Building on our scenario, changes might even relate to healthy or inflamed microenvironment.

In summary, cells of the myeloid lineage display genetic, qualitative, and quantitative changes in T1D. Yet, it remains unclear to what extend these differences contribute to onset of T1D.

## Therapeutic Opportunities

While a main goal of T1D research is to understand loss of immune tolerance, another objective is to restore tolerance in affected patients. Different therapeutic strategies aim on modulating cells of the myeloid lineage using granulocyte colony-stimulating factor (G-CSF) or granulocyte-macrophage colony-stimulating factor (GM-CSF). In presence of GM-CSF, cDCs can induce T_reg_ proliferation, while G-CSF increases levels of cDC2s and shifts the cytokine profile from T_H_1 toward T_H_2 in healthy individuals (86–88). Furthermore, G-CSF has an immune-regulatory effect, as indicated by increased levels of tolerogenic DCs (89, 90).

Clinical trials in T1D using colony-stimulating factor focused mainly on G-CSF, based on findings that G-CSF prevents diabetes in NOD mice by recruiting pDCs and functional CD4^+^CD25^+^T_regs_. Obtained T_regs_ protected against diabetes onset when transferred into NOD.*scid* mice (91). G-CSF treatment combined with anti-thymocyte globulin (ATG) reversed diabetes in NOD mice (92). In the clinic, ATG together with G-CSF preserved ß-cell function in T1D patients up to one year after treatment (93). However, a consecutive study indicated ATG as the main factor, because G-CSF alone increases numbers of circulating neutrophils, while C-peptide level or insulin needs remained unaffected. In addition, CD4:CD8 and naïve:memory T-cell ratios did not change upon G-CSF treatment (94). If anything, G-CSF even seemed to reduce the benefits of ATG. Patients treated either with ATG/G-CSF, or ATG alone had reduced conventional and regulatory CD4^+^ T-cell numbers after 2 weeks, with stable CD8^+^ T-cell numbers (95).

Since targeting myeloid hematopoiesis does not seem to offer major benefit to patients, other myeloid strategies might be more successful (96, 97). As discussed above, monocyte derived tolDCs do not differ between healthy and diseased individuals (98). Given their role as connectors and immune modulators, it seems plausible to use patients’ tolDCs to restore immune tolerance (99–101). In a recent clinical trial, tolDCs generated from monocytes by vitaminD3 followed by dexamethasone and loaded with proinsulin peptide C19-A3 were tested to restore immune tolerance in long-term T1D patients, demonstrating feasibility, safety, tolerability and mechanistic efficacy of this novel therapeutic intervention strategy engaging innate immunity (47). This strategy will next be tested for its capacity to delay disease progression and preserve endogenous beta-cell function.

## Conclusion

A myeloid footprint exists in pancreatic islets, irrespective of insulitis. Resident myeloid immune cells play a key role in islet morphology, physiology and function and are essential for tissue homeostasis and clearance of cell debris. These innate cells are intrinsic components in dialogue between islets and the immune system. Their role in diabetes seems clear in rodent models of autoimmune diabetes, but remains ignored, vague, inconsistent and inconclusive for human T1D. While limited information on residing myeloid cells in human islets is available after disease onset, our knowledge on these moderators before and during onset is even scarcer. Genetic variation and phenotypic differences in myeloid cells have been linked to T1D, but causality remains unclear. Subtle differences between health and disease can be largely attributed to dysglycemia, and may be a consequence, rather than causative, diabetogenic feature. Islet-resident myeloid immune cells conceivably prime the adaptive immune system, but with reason, as they are equipped to sense danger and tissue distress, and play a crucial role in tissue sensing, spatial antigen presentation, and tissue remodeling, in addition to immediately responding to ß-cell stress, changes in the microenvironment, or invading pathogens. Their failure in this process could predispose or trigger T1D. Dendritic cells can function as both sensors and connectors to the adaptive immune system. Adaptive immunity needs these cells to present islet autoantigens to the immune system so it is conceivable that they are involved in propagating the autoimmune response, while they could equally contribute to restoring/repairing islet tissue homeostasis, as well as restore immune tolerance! We contend that the innate immune system and myeloid cells in particular are connecting the dots in T1D. Their footprint in healthy islets underscores their essence and warrants more investigation. Therefore, it is critically important to learn more about changes between benign leukocyte residency and infiltration into pathogenic footprint and what causes these, to turn this knowledge into novel therapeutic intervention modalities and strategies. Engaging myeloid immune cells holds great promise as future treatment options.

## Data Availability Statement 

The original contributions presented in the study are included in the article/supplementary material. Further inquiries can be directed to the corresponding author.

## Author Contributions

Both authors studied literature and composed this review and its figures. Both authors contributed to the article and approved the submitted version.

## Funding

BR is director of the Wanek Family Project for Type 1 Diabetes.

## Conflict of Interest

The authors declare that the research was conducted in the absence of any commercial or financial relationships that could be construed as a potential conflict of interest.
